# Detection and molecular characterisation of bovine corona and toroviruses from Croatian cattle

**DOI:** 10.1186/s12917-015-0511-9

**Published:** 2015-08-13

**Authors:** Ivana Lojkić, Nina Krešić, Ivana Šimić, Tomislav Bedeković

**Affiliations:** Department of Virology, Croatian Veterinary Institute, Savska cesta 143, 10000, Zagreb, Croatia

**Keywords:** BCoV, BToV, Nucleocapsid, Spike, Phylogenetic analysis

## Abstract

**Background:**

Bovine coronavirus (BCoV) together with bovine torovirus (BToV), both members of the *Coronaviridae* family, order *Nidovirales* are the most common viral enteric pathogens. Although studied separately, their joint occurrence and the molecular diversity in cattle in Croatia have not been investigated.

**Methods:**

A survey is carried out on 101 fecal samples from diarrheic young and adult cattle during the 3-year period from i) one large dairy herd, ii) four small herds and iii) three nasal and paired fecal samples from calves with symptoms of respiratory disease. Samples were submitted to RT-PCR and sequencing for BCoV *Nucleocapsid* gene, BCoV *Spike* gene and BToV *Spike* gene.

**Results:**

BCoV was detected in 78.8 % of fecal samples from symptomatic cattle and three nasal and paired fecal samples from calves with respiratory symptoms. BToV was detected in 43.2 % of fecal samples from symptomatic cattle and a fecal sample from calves with respiratory symptoms. Molecular characterisation of those viruses revealed some nucleotide and aminoacid differences in relation to reference strains.

**Conclusions:**

BToV should be regarded as a relevant pathogen for cattle that plays a synergistic role in mixed enteric infections.

## Background

Diarrhea is an important disease affecting cattle worldwide. Together with the Bovine rotavirus (BRoV), bovine coronavirus (BCoV) and bovine torovirus (BToV), both members of the *Coronaviridae* family, order *Nidovirales* [1] are the most common viral enteric pathogens. They both cause diarrhea and respiratory-tract infections in calves as well as in adult cattle [2–5].

*Coronaviridae* members are enveloped viruses with non-segmented positive-sense single stranded RNA genome [6]. These viruses share the same basic genome organization and similar replication strategies. However, there are marked differences in genome size, host range, and virion architecture [1, 7] and there is no antigenic relationship between these two viruses. The virions of corona and toroviruses contain four and five structural proteins, respectively: the spike (S), the membrane (M), the haemagglutinin–esterase (HE) protein, the nucleocapsid (N) protein and the small envelope (E) protein [8]. The latter is not present in toroviruses. Another specificity of some coronaviruses is the internal nucleocapsid ORF coding for I protein [9]. Variations in host range and tissue tropism of coronaviruses are attributed to the spike (S) glycoprotein [10]. This protein is cleaved by an intracellular protease into two functional domains [10]. The peripheral S1 subunit is responsible for virus binding to host-cell receptors [11], induction of neutralizing antibodies [12], and haemagglutination activity [13]. The S1 sequence is variable, mutations in this region have been associated with altered antigenicity and virus pathogenicity [14] and this region has been exploited as a target to study the molecular epidemiology of BCoV infection [15, 16]. The sequence of the S2 subunit is more conserved and this subunit is responsible for cell membrane fusion activity [17].

BCoV infection has a high morbidity but a low mortality and is found worldwide among cattle of all ages [6]. Outbreaks typically occur in autumn and winter [18, 19]. Economic losses can be heavy due to a marked reduction in milk yield [20, 21].

BToV, formerly called Breda virus, was originally isolated from diarrheic calves in Breda, Iowa, in 1979. Until today, BToV was described in diarrhoeic calves in various countries [4, 2, 22–26, 27]. The faecal prevalence of BToV in calf diarrhea ranges from 2.9 % in South Korea [25] to 36.4 % in southern Ontario, Canada [22].

There are no available published data about the viral agents that are involved in calf diarrhea, although reports of the farmers complaining in calf diarhhea are very often. In this work we investigated fecal samples of cattle from one large dairy herd and from four small farms during the 3-year period. We also characterised nasal and paired fecal samples from calves with symptoms of respiratory disease. So for the first time, the occurrence and the molecular phylogeny of BCoVs and BToVs in selected herds from Croatia are assessed.

## Methods

### Ethics statement

This statement confirms that sampling for the purpose of this research was performed as non experimental clinical work with respecting the rules of veterinary profession. All samples were collected by veterinarian Nina Krešić, license number 2202. Sampling was performed strictly on the owner request. This statement is an annex to Ethical Committee permission of Veterinary Faculty University of Zagreb; number: 251-61-01/139-11-72.

### Samples

Fecal samples from 101 diarrheic animals were collected from March 2010 to May 2012. Samples originated from calves and adult cattle from one large dairy herd (designated as “B”) in eastern Croatia (*N* = 65) and four small family farms in Central Croatia (designated as “K”), all mixed dairy-beef production (*N* = 35). Three nasal swabs were collected in 2011 in east Croatia from calves showing respiratory symptoms (designated as “D”). A paired pooled fecal sample was taken from the same calves. All sampled cattle were showing clinical signs of enteric infection except those designated as D71-D73 where respiratory signs were present. Fecal samples were diluted 1:10 in minimal essential medium (pH 7.4; Life Technologies, USA) with addition of 1 % Antibiotic Antimycotic Solution (Sigma- Aldrich). Suspensions were centrifuged at 12,000 g for 15 min at 4 °C and only the supernatants were used in the assays. All fecal samples were also tested by RT-PCR for rotavirus-A [27]. Nasal samples were tested by PCR/RT-PCR for bovine herpes virus type 1 (BHV-1) [28], bovine respiratory syncytial virus (BRSV) [29], bovine parainfluenza virus type 3 (BPIV-3) (in-house method) and bovine viral diarrhea virus (BVDV) [30].

### cDNA synthesis and PCR

Viral RNA was extracted from samples using QIAamp®Viral RNA (QIAGEN, Hilden, Germany), according to manufacturer's instructions. cDNA synthesis were performed with Moloney-Murine leukaemia virus reverse transcriptase (M-MLV RT) (Invitrogen, USA) and random primers (50 ng/lL) (Invitrogen) in a 20 uL final reaction volume. The cDNA of each sample was screened for the BCoV, BToV and BRoV-A genome using the primers described in Table 1. PCR reactions were performed using GoTaq™ Green Master Mix (Promega, USA), according to manufacturer instructions. A total of 16 samples were chosen for sequencing; all samples were sequenced for partial BCoV N and S gene, and five of these samples were sequenced for BToV S gene as well. Eight samples were from large dairy farm in eastern Croatia (B27/10, B30/10, B32/11, B37/11, B3492/11, B34649/11, B60853/11, B6075/12); four samples were from small family farms in Central Croatia (K12/10, K658/10, K5220/11, K6578/11), and four from calves with respiratory symptoms (D71-D73F). Amplified PCR products were purified using Exosap (USB, Staufen, Germany) and direct sequenced using the PCR primers in both directions by Macrogen Inc. (Seoul, Korea). Nucleotide sequences generated in this study have been submitted to GenBank and were assigned the following accession numbers as listed in Table 2.

**Table 1:** Oligonucleotide primers for PCR and sequencing reaction

PRIMER NAME	SEQUENCE (5’ → 3’)	SIZE (bp) and location of amplicon	GENOME POSITION^a^	REFERENCE
BCoV F	CCGATCAGTCCGACCAATC	460	29476–29494	[46]
BCoV R	TAGTCGGAATAGCCTCATCGC	BCoV N gene	29899–29919	
S-S1	GATAAGTTTGCTATACCCAATGG	1194	24817–24839	[47]
S-AS1	ACTATCATTTACTGAATTAACAG	BCoV S gene	25988–26010	
TORO S5	GTGTTAAGTTTGTGCAAAAAT	741	20956–20976	[5]
TORO S3	TGCATGAACTCTATATGGTGT	BToV S gene	21677–21697	

^a^Numbering according to the complete genome of BCoV strain Mebus (U00735) and BToV strain Breda 1 (AY427789)

**Table 2:** Accession numbers of Croatian BCoV partial N and S and BToV partial S gene sequences

Isolate name	BCoV N Accession no	BCoV S Accession no	BToV S Accession no
B 27/10^a^	KM677147	KM677163	KM677179
B30/10^a^	KM677148	KM677164	
B32/11^a^	KM677149	KM677165	
B34649/11^a^	KM677150	KM677166	
B3492/11	KM677151	KM677167	KM677182
B37/11	KM677152	KM677168	
B6075/12^a^	KM677153	KM677169	KM677180
B60853/11^a^	KM677154	KM677170	KM677181
D71/11	KM677155	KM677171	
D72/11	KM677156	KM677172	
D73/11	KM677157	KM677173	
D71-73 F	KM677158	KM677174	KM677183
K12/10^a^	KM677159	KM677175	
K5220/12^a^	KM677160	KM677176	
K6578/11^a^	KM677161	KM677177	
K658/10	KM677162	KM677178	

^a^Those samples were also RT-PCR positive to BRoV-A

### Sequence alignment and phylogenetic analysis

Sequences were aligned and compared to previously published BCoV N, BCoV S and BToV S sequences, respectively. Sequence identities of nucleotides as well as estimation of the evolutionary divergence between sequences were analyzed using BioEdit and Mega6 [31] software, respectively. The neighbour-joining (NJ) trees were obtained using Mega6 program with the evolutionary model set to Tamura-Nei + Gamma for Spike gene analysis and Kimura-2 parametar for N gene. Estimation of best-fit model by hierarchical likelihood ratio tests (hLRTs) and approximate Akaike information criterion (AIC) was performed with jModelTest V.0.1.1. [32]. Reliabilities of phylogenetic relationships were evaluated using nonparametric bootstrap analysis with 1000 replicates for NJ analysis. Bootstrap values exceeding 70 were considered well supported.

The dataset included the reference strains of BCoV: Mebus (GenBank accession number U00735), Quebec (AF220295) respiratory BCoV strains: LSU2 (AF058943), OK-3 (AF058944) and AH187 (FJ938065); Italian strains 179/07–11 (EU019216), 438/06-TN (EU814647) and 339/06 (EF445634); Korean strain KWD19 (DQ389660), Japanese strain Kakegawa (AB354579), Swedish strain SWE/I/08-3 (KF169933), Danish strain DEN03-2 (KF169914), Irish strain RVLC9 (KF272913), Human coronaviruses: Hu-OC43 (Z32769) and Hu-4408 (L07748; FJ415342). Reference strains of BToV: Breda1 (AY427798), NA7 (AB254073), Gifu-2007TI/E (AB526863), Hokkaido-2008TI/E (AB526864), Aichi/2004 (AB526866) and B145 (AJ575373).

## Results

From 101 analyzed fecal and three nasal samples, 82 were positive for BCoV (78.8 %), and 45 for BToV (43.2 %). 36 samples were positive for BRoV + BCoV; 30 for BCoV + BRoV + BToV; 11 for BCoV + BToV, 4 for BRoV + BToV. Five samples were positive for BCoV only and 11 to BRoV only. From 16 samples chosen for sequencing, 37 sequences were obtained (Table 2). Samples were chosen to cover three-year period.

### BCoV N gene

**Fig. 1:**
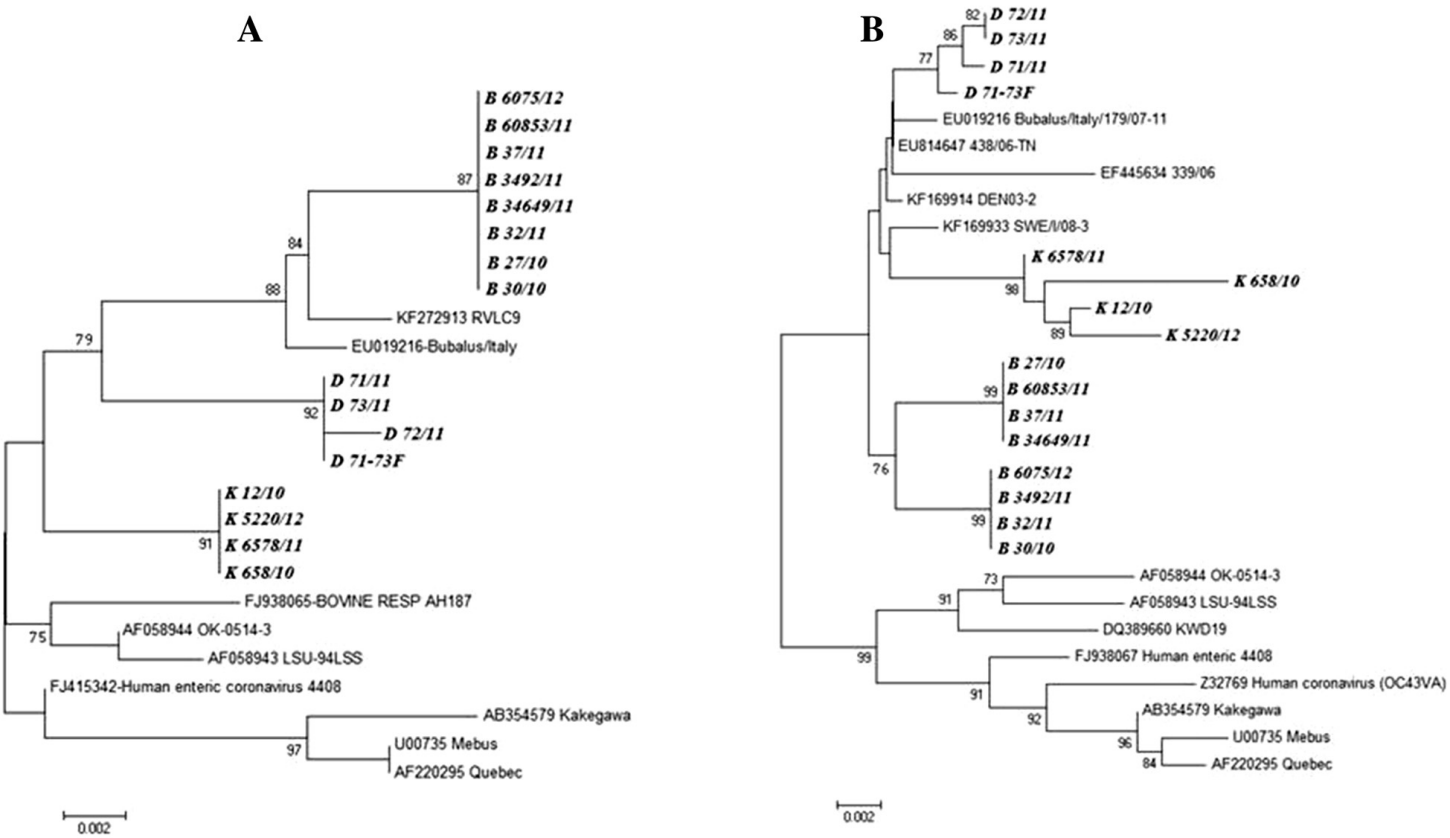
Neighbour-joining phylogenetic trees of 362-nt BCoV N gene sequences (**a**) and 975-nt BCoV S gene sequences (**b**) from 16 Croatian and 13 sequences form Genbank. Strains in the tree are shown by accession number and name. BCoV isolates from Croatia are shown in bold italics. Numbers on nodes indicate bootstrap support (*n* = 1000). The bar at the bottom of the figure denotes distance

Comparative analysis of the BCoV N sequences (nucleotides (nt) 124–485; N protein amino acid (aa) 42–165; I protein aa 21–142) showed that all Croatian strains obtained in 2010–2012 shared a high identity both at the nt level (98–100 %) and at the deduced N protein aa level (98.3–100 %). Sequences from eight samples from large dairy farm designated as “B” in the Eastern region were mutually 100 % identical and most similar to Italian strain 179/07–11 Bubalus (EU019216) and Irish RVLC9 (KF272913) (99,1 %). Fecal samples designated as “K” and fecal and nasal samples “D” were more similar to human enteric isolates OK-0514-3 and 4408 (FJ415342) (98.8–99.1 %) but phylogenetically clustered with other Croatian strains and to Italian 179/07–11 and Irish RVLC9 (Fig. 1a). The BCoV strains obtained from respiratory and enteric disease did not show any consistent nucleotide differences in the sequenced N region.

### BCoV S gene

The BCoV strains obtained from respiratory disease showed two aa differences in the sequenced S1 region: (408 Ser-Ile, 470 Ile-Val).

Comparative analysis of the 975 nt BCoV S sequence (nt 1258–2230; aa 387–710) showed that all Croatian strains shared a high identity both at the nt level (97.7–100 %) and at the deduced aa level (97.5–100 %). “K” sequences were identical 98.6–99 %, “B” 99–100 % and “D” 99.6–100 %.

Our isolates were most similar to Danish strain DEN03-2 (KF169914) (99.1–99.6 %) and Italian strains Bubalus 179/07–11, 438/06-TN and 339/06 (99.0–99.3 %) and were phylogenetically clustered with them (Fig. 1b). On aa level, our isolate K12 were 100 % identical to Danish DEN03-2 (KF169914).

### BToV S gene

**Fig. 2:**
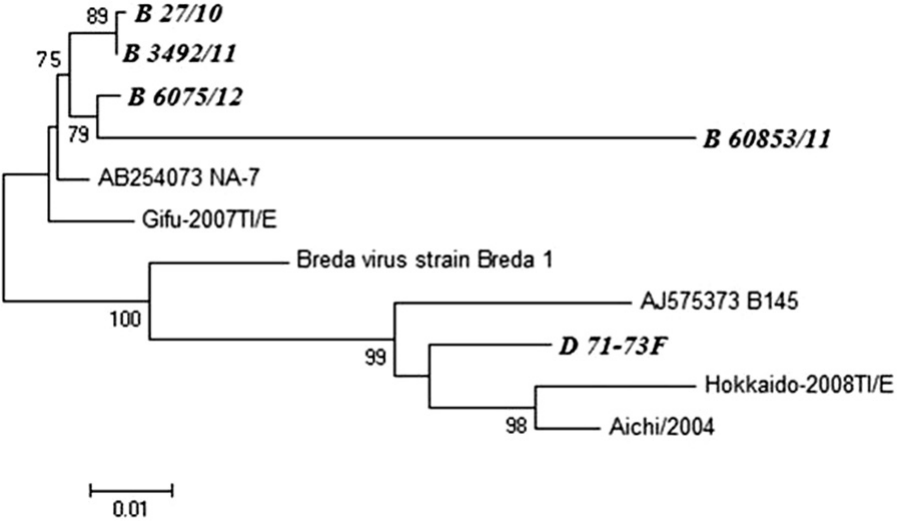
Neighbour-joining phylogenetic tree of 608-nt BToV S gene sequences from 5 Croatian and 6 sequences form Genbank. Strains in the tree are shown by accession number and name. BToV isolates from Croatia are shown in bold italics. Numbers on nodes indicate bootstrap support (*n* = 1000). The bar at the bottom of the figure denotes distance

Analysis of the 608 nt BToV S sequence (nt 65–671; aa 22–222) showed 93.2–99.8 % identity between our strains at the nt level and 83.7–99.5 % at the deduced aa level (except for the strain B60853/11 that were more divergent). Croatian BToV strains “B” did not show any consistent nt or aa differences in the sequenced S region. They were most similar to Japanese NA-7 (AB254073) (93.4–99.0 %) and were phylogenetically clustered with them (Fig. 2). Isolate D71F, obtained from the feces of calf with respiratory symptoms were most identical to Japanese Aichi (96.5 %) and European B145 (96 %).

## Discussion

This is the first report on the detection of torovirus infection in cattle, as well as molecular characterization of BCoVs and BToVs in Croatia. We determined the partial N and S gene sequences of BCoV and partial S gene sequences of BToV from i) one large dairy herd during 3-year period (“B”), ii) four small family farms (“K”) and iii) paired fecal and nasal samples from calves entered into feedlot (“D”). BCoV vaccination is not practiced in Croatia.

All Croatian BCoV N and S gene sequences are clustered together and with sequences from Italy, Denmark and Sweden, separately from the cluster with respiratory strains and the reference strain Mebus (Fig. 1). In this cluster they were further grouped geographically as “B”, “K” or “D” subgroup. The similar clustering is obtained when aa sequences for N and I proteins were analysed (data nor shown). All sequences from the same subgroup were mutually identical when N gene was analysed. More diversity is recognized when S gene was analysed, but sequences from large dairy herd (“B”) were still 99–100 % identical. This lead to the conclusion that the same BCoV strain circulated for extended period of time within one herd. In our investigation, we covered the S region from aa 387–701 which covers the previously reported hypervariable region (aa 452–593) [33, 34] but not the S cleavage site (aa 764–768). Samples obtained from small family farms (“K”) showed less mutual identity on S (98.6–99 %) compared with those that came from the same herd (“B”). The BCoV strains obtained from respiratory disease showed two aa differences in the sequenced S region: (408 Ser - Ile, 470 Ile - Val), but compared to paired fecal sample, no aa differences and only two nt differences were found. Previous researchers demonstrated that some BCoV strains isolated from the respiratory tract had different biological, antigenic [35–37] and genetic [38, 39] properties compared with enteric BCoV strains, whereas others did not detect any consistent differences [40, 41] and some suggested that the same strains of BCoV cause natural outbreaks of respiratory and enteric disease [20, 21, 42, 43]. Our findings, although based on a minimal data are in agreement with the latter fact; there were no differences between respiratory/fecal sample pair. Still, the identified change in amino acid 408 could affect the aa polarity (hydrophilic to hydrophobic).

Regarding the herd designated as “D”, outbreak of respiratory disease followed soon after entry into a feedlot. Those calves were also RT-PCR positive to BRSV but negative to BHV-1, BPIV-3 and BVDV. Therefore, it was a typical scenario of Bovine respiratory complex (BRC) as a consequence of stress from transport and change in husbandry.

The percentage of BToV infections in the present study (43.2 %) is higher than that reported in South Korea - 2.9 % [25], Lower Saxony, Germany-5 % [44], Japan- 8.4 % [26], USA- 9.7 % [5] and Canada- 36.4 % [22]. Still, conclusions based on these frequencies are not reliable because this study was not originally planned as an epidemiological study. According to previous study [45], BToV alone has been shown to act as a primary enteric pathogen in cattle. In our study, BToV was found only in co-infection with BCoV and BRV-A (Table 2). It is noteworthy to say that co-infection with BRoV-A had not influence on BCoV and BToV sequence diversity. Despite the fact that BToV causes diarrhea and respiratory infections in cattle of all ages [6] our isolates from respiratory disease were negative. This also means that respiratory BToV infection cannot be excluded if more samples has been analysed. Croatian BToVs showed moderate to high degree of nucleotide (87.1–99.8 %) and amino acid identity (83.7–99.5 %). Comparison of our sequences with one available BToV S gene sequence from Europe (AJ575373*)* and four sequences from Japan showed high degree of sequence identity (93.4–99.0 %) between our strains from large dairy farm (“B”) and Japanese strain NA-7 (AB254073). On contrary, sequence from feces of calves with respiratory symptoms were more similar to European strain B145 (AJ575373) (96 %), and Japanese strain Aichi (96.5 %). Phylogenetic analysis revealed that our sequences of BToV S gene from symptomatic animals are more closely related to sequences from Japan than to the Breda 1 strain and European strain (Fig. 2). Still, fecal isolate from herd with respiratory disease clustered with European and other Japanese strains and was more related to Breda 1 (Fig. 2).

## Conclusions

Despite relatively small number of samples investigated, we can clearly conclude that BCoVs and BToVs (together with rotaviruses) are common enteric pathogens of cattle of all ages and production categories in Croatian herds. Required steps in herd management should include raising awareness of the adequate and timely colostrum intake by the calf as well as vaccination of the cows with one of the commercially available vaccine before calving. Although on minimal data, we have proved that the same BCoV strain circulated for extended period of time within one herd but different strains circulated in different herds. Molecular characterisation of those viruses revealed some nt and aa differences in relation to reference strains, but generally, Croatian sequences are clustered with other, mainly European BToV and BCoV isolate sequences.
